# Increasing Gram-Negative Catheter-Related Bloodstream Infection in Cancer Patients

**DOI:** 10.3390/pathogens12020228

**Published:** 2023-02-01

**Authors:** Julia Laporte-Amargos, Enric Sastre, Alba Bergas, Helena Pomares, Annalisa Paviglianiti, Marisol Rodriguez-Arias, Natalia Pallares, Ana Maria Badia-Tejero, Paula Pons-Oltra, Jordi Carratalà, Carlota Gudiol

**Affiliations:** 1 Infectious Diseases Department, Bellvitge University Hospital, Bellvitge Biomedical Research Institute (IDIBELL), University of Barcelona, L’Hospitalet de Llobregat, 08908 Barcelona, Spain; 2 Centro de Investigación Biomédica en Red de Enfermedades Infecciosas (CIBERINFEC), Instituto de Salud Carlos III, 28029 Madrid, Spain; 3 Hematology Department, Institut Català d’Oncologia (ICO), Hospital Duran i Reynals, IDIBELL, L’Hospitalet de Llobregat, 08908 Barcelona, Spain; 4 Oncology Department, ICO, Hospital Duran i Reynals, IDIBELL, L’Hospitalet de Llobregat, 08908 Barcelona, Spain; 5 Biostatistics Unit, IDIBELL, L’Hospitalet de Llobregat, 08908 Barcelona, Spain; 6 ICO, Hospital Duran i Reynals, IDIBELL, L’Hospitalet de Llobregat, 08908 Barcelona, Spain

**Keywords:** catheter-related bloodstream infection, port reservoir, onco-hematological patients, hematological malignancies, solid tumors, gram-negative

## Abstract

**Background**: We aimed to assess the incidence, etiology and outcomes of catheter-related bloodstream infection (CRBSI) in onco-hematological patients, to assess the differences between patients with hematological malignancies (HMs) and solid tumors (STs) and to identify the risk factors for Gram-negative (GN) CRBSI. **Methods**: All consecutive episodes of BSI in adult cancer patients were prospectively collected (2006–2020). The etiology of CRBSI was analyzed in three different 5-year periods. Risk factors for GN CRBSI were assessed in the whole cohort and separately in patients with HMs and STs. **Results**: Among 467 episodes of monomicrobial CRBSI, 407 were Gram-positive (GP) (87.1%), 49 GN (10.5%) and 11 fungal (2.4%). Hematological patients (369 episodes) were more frequently neutropenic and were more likely to carry central venous catheters and develop GP CRBSI. Patients with STs (98 episodes) had more comorbidities, more frequently carried port reservoirs and commonly presented more GN CRBSI. GN CRBSI significantly increased over the study period, from 5.2% to 23% (*p* < 0.001), whereas GP CRBSI decreased from 93.4% to 73.3% (*p* < 0.001). CRBSI episodes involving port reservoirs and peripherally-inserted central catheters were significantly increased (*p* < 0.001). The most frequent GPs were coagulase-negative staphylococci (CoNS) (57.8%) and *Pseudomonas aeruginosa* was the most common GN (3%). Multidrug-resistant (MDR) GN represented 32.7% of all GN CRBSIs and increased over time (*p* = 0.008). The independent risk factors for GN CRBSI in the whole cohort were solid tumor, chronic kidney disease and carrying a port reservoir. Carrying a port reservoir was also a risk factor in patients with STs. Health-care acquisition was identified as a risk factor for GN CRBSI in the whole cohort, as well as in patients with STs and HMs. Inadequate empirical antibiotic treatment (IEAT) occurred regardless of the etiology: 49% for GNs and 48.6% for GPs (*p* = 0.96). In GP CRBSI, IEAT was mainly due to inadequate coverage against CoNS (87%), whereas in GN CRBSI, IEAT was associated with multidrug resistance (54.2%). Early (48 h and 7-day) and 30-day case-fatality rates were similar when analyzed according to the type of underlying disease and etiology, except for the 30-day case-fatality rate, which was higher in the group of patients with STs compared to those with HMs (21.5% vs. 12.5%, *p* = 0.027). The 48 h case-fatality rate was significantly higher in patients in whom the catheter had not been removed (5.6% vs. 1%; *p* = 0.011), and it remained significant for GP CRBSI (6% vs. 1.3%, *p* = 0.023). **Conclusions**: GNs are an increasing cause of CRBSI in cancer patients, particularly in solid tumor patients carrying port reservoirs. Multidrug resistance among GNs is also increasing and is associated with higher rates of IEAT. Decreased 48 h survival was associated with the non-removal of the catheter. These findings should be considered when deciding on early therapeutic management for cancer patients with suspected CRBSI.

## 1. Introduction

The use of intravascular catheters is increasing in cancer patients in inpatient and outpatient settings for administering medication, chemotherapy and other relevant products. Nevertheless, this improvement in the management of cancer patients in modern-day medical practice is associated with a risk of infection. In this regard, catheter-related bloodstream infection (CRBSI) is one of the most frequent complications in cancer patients carrying intravascular catheters and is associated with increased morbidity and mortality, prolonged length of hospital stay and excess costs [1,2,3].

The use and management of intravascular catheters in cancer patients have changed in the last decade, with the increasing use of peripherally-inserted central venous catheters (PICCs), tunneled catheters and port reservoirs, and a decrease in peripheral and central venous catheters (CVCs). These changes, along with the recent changes in the epidemiology of bloodstream infection (BSI) and the wide implementation of preventive interventions, have led to a shift in the etiology of CRBSI. Even though Gram-positive (GP) bacteria, mainly coagulase-negative staphylococci (CoNS) and *Staphylococcus aureus*, remain the leading cause of CRBSI in cancer patients, a rise in Gram-negatives (GNs) as causative agents have been recently documented in some institutions [4,5,6]. In addition, the emergence of antibiotic resistance among GN-causing BSI in this population is particularly worrisome, because delayed adequate empirical antibiotic therapy is associated with increased mortality [6,7,8,9].

Current information on the epidemiology of CRBSI in onco-hematological patients according to the underlying malignancy is lacking [5]. Most studies have analyzed patients with solid tumors (STs) and patients with hematological malignancies (HMs) together [1,6,10], whereas these two patient groups are inherently different. The clinical features, site of CRBSI acquisition and type of catheter involved vary according to the underlying disease, and this may influence the potential CRBSI etiology and should influence the subsequent approach and management. In this regard, the most widely recognized guidelines for the management of CRBSI only consider coverage against GNs in neutropenic patients [2,11], and they were written in an era when the emergence of antibacterial resistance was not as high as we are currently facing. In addition, the different potential approach according to the type of underlying malignancy and type of catheter is not considered. Thus, the optimal empirical antibiotic therapy and management of cancer patients with suspected CRBSI has yet to be established.

The aim of this study is to assess the incidence, etiology and outcomes of CRBSI in onco-hematological patients during a 15-year period, to assess the differences between patients with STs and HMs and identify the risk factors for GN CRBSI.

## 2. Methods

### 2.1. Setting, Study Population and Study Design

This study was performed at a 200-bed university referral cancer center hospital for adults in Barcelona, Spain. All onco-hematological patients, including hematopoietic stem cell transplant recipients, with BSI, were included (January 2006–December 2020). Data were prospectively collected and retrospectively analyzed.

Information on baseline characteristics, clinical features, etiology, empirical antibiotic therapy and outcomes were collected in a database, as part of the standard infectious disease management at our hospital. The only exclusion criterion was polymicrobial etiology. Monomicrobial CRBSI episodes were analyzed and compared in three periods of 5-years (2006–2010, 2011–2015 and 2016–2020). Risk factors for GN CRBSI were assessed in the whole cohort, and also separately in the two groups of patients with HMs and with STs. For this purpose, GN episodes were compared with those caused by GP. The study was approved by The Clinical Research Ethics Committee and Institutional Review Board of Bellvitge University Hospital.

### 2.2. Definitions

CRBSI was defined according to the guidelines from the Infectious Disease Society of America (IDSA) [11]: Bacteremia or fungemia in a patient with an intravascular device and ≥1 positive blood culture result obtained from the peripheral vein, clinical manifestations of infection (e.g., fever, chills and/or hypotension) and no apparent source of BSI (with the exception of the catheter). One of the following should be present: a positive result of semi-quantitative (>15 colony-forming units [CFUs] per catheter segment) or quantitative (>10^2^ CFUs per catheter segment) catheter culture, whereby the same organism (species) is isolated from the catheter segment and the peripheral blood culture; simultaneous quantitative cultures of blood with a ratio of 13:1 CFUs/mL of blood (catheter vs. peripheral blood); differential time to positivity (growth in a culture of blood obtained through a catheter hub is detected using an automated blood culture system at least 2 h prior than that of a culture of simultaneously drawn peripheral blood of equal volume).

Polymicrobial BSI was defined when two or more organisms were isolated from blood culture specimens collected from a patient during a period of <72 h. Neutropenia was defined as an absolute neutrophil count <500/mm^3^. Septic shock was defined as a systolic blood pressure <90 mmHg which was unresponsive to fluid treatment or required vasoactive drug therapy. The Multinational Association for Supportive Care in Cancer (MASCC) score was calculated in patients with a neutrophil count of <500/mm^3^ or <1.000/mm^3^ and expected to fall below 500/mm^3^ within 24 to 48 h, as described elsewhere [12]. The MASCC score categorizes the episodes of febrile neutropenia according to the risk of presenting complications (low risk vs. high risk).

Initial empirical antibiotic therapy was considered inadequate if the treatment regimen did not include at least one antibiotic active in vitro against the infecting microorganism. GN bacilli were considered to be multidrug-resistant (MDR) in the following situations: (a) extended-spectrum beta-lactamase (ESBL)-producing Enterobacterales; (b) AmpC-cephalosporinase hyper-producing Enterobacterales; (c) carbapenem-resistant Enterobacterales (d) microorganisms with intrinsic resistance mechanisms, such as *Stenotrophomonas maltophilia* and (e) MDR strains, including *Pseudomonas aeruginosa* and *Acinetobacter baumannii* as defined by Magiorakos et al. [13].

### 2.3. Microbiologic Studies

Two sets of two 8–10 mL blood samples (Bactec Plus Aerobic and Anaerobic, BD), were taken 30 min apart from all patients who presented with fever ≥ 38 °C or when bacteremia was suspected by clinical signs or symptoms. Blood samples were processed in a BACTEC 9240 (from 2006 to 2010) or a BACTEC-FX (since May 2010) apparatus (BD Microbiology Systems) with an incubation period of 5 days. Positive blood samples were subcultured onto chocolate agar. Identification and antibiotic susceptibility of GN bacilli, *Enterococcus* spp. and *Staphylococcus aureus* were performed using commercially available panels MicroScan from the Walkaway automated system (BeckmaneCoulter). Identification of other *Streptococcus* spp. was performed using standard biochemical testing and antibiotic susceptibility with commercial panels Sensititre (TREK Diagnostic System). Anaerobes identification was performed using standard biochemical testing and antibiotic susceptibility with the E-test method (BioMérieux). In addition, since November 2012, identification has been performed using Matrix-Assisted Laser Desorption Ionization (MALDI-TOF; Bruker Daltonics). The European Committee on Antimicrobial Susceptibility Testing (EUCAST) recommendations and criteria was used to define susceptibility or resistance to antimicrobial agents [14].

### 2.4. Statistical Analysis

Continuous variables were compared using the Mann-Whitney U test and the Student t test, as appropriate. Qualitative variables were compared using the chi-square test. Odds ratios (ORs) and 95% confidence intervals (CIs) were calculated. A *p* value of < 0.05 was considered statistically significant.

Multivariate conditional logistic regression analysis of factors potentially associated with GN CRBSI included all statistically significant variables in the univariate analysis, along with sex, age and all clinically important variables regardless of whether they were statistically significant. The analysis was performed with the stepwise logistic regression model of the SPSS software package v. 17.0 (SPSS Inc., Chicago, IL, USA).

## 3. Results

### 3.1. Study Population

A total of 3013 episodes of BSI in onco-hematological patients were documented during the study period. Among them, 503 (16.7%) were CRBSI. Thirty-six polymicrobial episodes (7.15%) were excluded from the analysis, which left a total of 467 episodes of CRBSI. Table 1 shows the baseline characteristics, clinical presentation and outcomes of patients with CRBSI according to the underlying disease (369 episodes in patients with HMs and 98 occurring in patients with STs). The most frequent hematological malignancy was acute leukemia (56%) followed by lymphoma (22.8%), whereas breast cancer (24.4%) and colorectal cancer (23.4%) were the most frequent solid neoplasms. Hematological patients were more frequently neutropenic (57.6% vs. 11.2%, *p* < 0.001) and were more likely to carry central venous catheters (82.9% vs. 18.4%, *p* < 0.001), whereas patients with solid tumors had more comorbidities (25% vs. 36.7%, *p* = 0.020), and more frequently carried other type of catheters, particularly port reservoirs (1.1% vs. 45%, *p* < 0.001). GP CRBSI was significantly higher in hematological patients (90% vs. 76.5%, *p* < 0.001), whereas GN CRBSI occurred more frequently in patients with solid tumors (8.7% vs. 18.4%, *p* = 0.004). Outcomes of patients did not differ significantly, except for the overall 30-day case-fatality rate, which was significantly higher in patients with STs (12.5% vs. 21.5%, *p* = 0.027).

### 3.2. Rates of CRBSI

Among the 467 episodes of CRBSI, 407 were GP (87.1%), 49 GN (10.5%) and 11 fungal (2.4%) Table 2 shows the evolution of the CRBSI rates, etiology and types of catheters involved in the different time periods. The number of CRBSI episodes decreased through the study period from 183 to 116 episodes. GN BSI significantly increased from 5.4% to 22.4% over the study period (*p* < 0.001), whereas GP BSI decreased from 93.4% to 73.3% (*p* < 0.001). The great majority of CRBSI episodes involved central venous catheters (CVCs) placed predominantly in the subclavian vein (69.3%). The type of catheter implicated varied through the study period: CRBSI episodes involving peripheral catheters significantly decreased over time, whereas episodes involving port reservoirs and peripherally-inserted central catheters increased (*p* < 0.001).

### 3.3. Etiology

The etiology of the 467 episodes of monomicrobial CRBSI episodes is displayed in Table 3. GP CRBSI accounted for the great majority of episodes (87.1%), with CoNS being the most frequent organisms (57.8%; 208 out of 268 identified as *Staphylococcus epidermidis* (77.6%)), followed by *S. aureus* (22.8%) and *Enterococcus* spp. (4.1%). Among GNs (10.5%), *P. aeruginosa* was the most common GN (3%), followed by *Klebsiella* spp. (2.4%). Eleven episodes were caused by *Candida* spp., being non-albicans species predominant (81.8%), and led by *C. parapsilosis* (45.5%). Among GNs, the rate of multidrug resistance significantly increased over time, from 2.2% to 8.7% (*p* = 0.08). The most frequent MDR GN were ESBL-producing Enterobacterales (n = 6, 35.2%), followed by *S. maltophilia* (n = 5, 29.4%) and carbapenemase-producing Enterobacterales (n = 3, 17.6%).

### 3.4. Risk Factors for GN CRBSI

The differences between episodes caused by GN and GP are shown in Table 4. Patients with GN CRBSI had frequently more STs (36.7% vs. 18.4%; *p* = 0.005), chronic kidney disease (10.2% vs. 2%; *p* = 0.008), higher risk according to the MASCC risk score (53.3% vs. 27.5%; *p* = 0.036) and port reservoirs (26.5% vs. 8.1%; *p* < 0.001). GP CRBSI predominated in hematological patients (63.3% vs. 81.6%, *p* = 0.005) with neutropenia (27.1% vs. 51.4%, *p* = 0.002) and during hospitalization (76.4% vs. 38.8%, *p* < 0.001).

In the multivariate analysis, the risk factors associated with GN CRBSI in the whole cohort were STs as the underlying disease (OR 2.57; 95% CI 1.34–4.82, *p* = 0.005), chronic kidney disease (OR 25.69; 95% CI 1.61–18.2, *p* = 0.009) and to carry a port reservoir (OR 4.09; 95% CI 1.92–8.38, *p* < 0.001). Carrying a port reservoir was also a risk factor for GN CRBSI in patients with STs (OR 4.00; 95% CI 1.34–13.9, *p* = 0.012). The health-care acquisition was identified as a risk factor for GN CRBSI in the whole cohort, as well as in patients with STs and HMs: (OR 5.08; 95% CI 2.75–9.60, *p* < 0.001), (OR 3.05; 95% CI 1.02–10.6, *p* = 0.045) and (OR 5.24; 95% CI 2.44–11.4, *p* ≤ 0.001), respectively.

### 3.5. Outcomes

Overall, inadequate empirical antibiotic treatment (IEAT) was observed in 232 patients (49.7%), and the reason was not administering any antibiotic empirically in 80 of them (31.7%). IEAT occurred regardless of the etiology: 49% in GN CRBSI vs. 48.6% in GP CRBSI, (*p* = 0.96). In GP CRBSI, IEAT was mainly due to inadequate coverage against CoNS (87%), whereas in GN CRBSI, IEAT was associated with multidrug resistance (54.2%). IEAT was not associated with increased 48 h, 7-day or 30-day case-fatality rates (2% vs. 2%, *p* = 0.75; 5.6% vs. 5.6%, *p* = 0.99 and 16.5% vs. 13%, *p* = 0.26, respectively), including when analyzed separately according to the etiology.

The overall 48 h, 7-day and 30-day case-fatality rates were 2%, 5.6% and 14.5%, respectively. These outcomes were similar when analyzed according to the type of underlying disease and etiology, except for the 30-day case-fatality rate, which was higher in the group of patients with STs compared to those with HMs (Table 1 and Table 4). The 48 h case-fatality rate was significantly higher in patients in whom the catheter had not been removed (5.6% vs. 1%; *p* = 0.011), and it remained significant for GP CRBSI (6% vs. 1.3%, *p* = 0.023).

## 4. Discussion

In this study of a large cohort of onco-hematological patients with CRBSI, we found that the overall rate of CRBSI decreased over time, the rate of BSI due to GNs and MDR GNs increased along with the use of port reservoirs and PICCs and that 48 h mortality was higher in patients in whom the catheter had not been removed. We also observed some differences between the episodes occurring in patients with HMs and STs and identified different risk factors for GN CRBSI in these two groups of patients.

Our findings showed a decrease in the number of CRBSI in cancer patients through a 15-years study period. This could be explained by the implementation of a specific program to prevent the development of CRBSI in hospitalized patients in our institution in the last decades. In this regard, it has been shown that the implementation of interventions and bundles to prevent CRBSI in cancer patients is cost-effective [2]. We also observed a shift towards GNs as an emerging cause of this infectious complication. Similar results have been previously published in the immunocompromised population with cancer [1,4,5,6]. A plausible explanation for this changing epidemiology is the increased management of cancer patients in the outpatient setting, using PICCs and port reservoirs. The physiopathology of CRBSI in this scenario is different from hospitalized patients with CVCs, in which the colonization of the catheter hubs by common skin GP colonizers through the hands of the health personnel is the first step to catheter infection [15,16]. In the outpatient setting, the catheter infection could be more related to the suboptimal care of the device. In addition, in patients with port reservoirs, the catheter tip could be secondarily colonized in patients with BSI from the gastrointestinal tract. In this line, some reports have also identified GNs as the main cause of CRBSI in patients with malignancies carrying Hickman catheters and PICCs [10,17].

Of note, MDR GNs also increased during the study period, with ESBL-producing Enterobacterales being the most frequently resistant GNs. Remarkably, *S. maltophilia* mainly developed in hematological patients (83.3%) and complicated with alveolar hemorrhage in three patients (50%), with fatal outcomes in two (33.3%). Although it is not very frequent, CRBSI due to *S. maltophilia* is associated with very poor outcomes [18]. Additionally, all carbapenemase producers were isolated in the last study period, which agrees with the increase in carbapenem resistance worldwide [19,20]. Finally, the rate of fungal BSI has slightly increased over time, with a clear predominance of non-albicans *Candida* spp. These results are in line with several reports involving cancer patients with candidemia [21,22].

Data regarding the different features of CRBSI according to the underlying malignancy is particularly scarce [6]. In our cohort, we observed that hematological patients were more frequently neutropenic and presented CRBSI mainly due to CoNS due to CVC colonization during hospitalization. Conversely, GN CRBSI in patients with STs occurred commonly in the outpatient setting and often involved port reservoirs and PICCs. Even though the rate of IEAT was high in all groups of patients, it was not associated with increased mortality. This could be explained, on the one hand, because in hematological patients, IEAT was mainly due to CoNS BSI, which has been shown not to be associated with mortality. On the other hand, the catheter was promptly removed in all patients with infection with resistant organisms. In this line, in patients with CRBSI due to particular etiologies (such GNs, *S. aureus* and fungi), the source control has been shown to be indispensable for favorable outcomes [11,23,24]. In fact, the only modifiable variable identified with survival in our cohort was catheter removal.

In the whole cohort, the independent risk factors for GN CRBSI were STs, chronic kidney disease and port reservoirs, and in patients with STs, carrying a port reservoir was identified as a main risk factor. In addition, health-care acquisition was found to be a risk factor in the three groups of patients. These results support the hypothesis that outpatient management, mainly in patients with STs, and using port reservoirs, are the main variables associated with GN CRBSI.

This study has some limitations that should be acknowledged. First, it was a single-center study, which hinders the generalization of the results, because microbiological epidemiology varies significantly in different geographical areas. Second, we couldn’t provide data on the time from catheter insertion to CRBSI. However, the strengths of the study are the large number of patients included in the analysis and the prospective collection of the data.

In conclusion, GNs are an increasing cause of CRBSI in cancer patients, particularly in patients with ST-carrying port reservoirs. Multidrug resistance among GNs is also increasing and is associated with higher rates of IEAT. Decreased 48 h survival was associated with non-removal of the catheter. These findings should be considered when deciding on early therapeutic management of cancer patients with suspected CRBSI.

## Figures and Tables

**Table 1:** Baseline characteristics, clinical presentation and outcomes of patients with catheter-related bloodstream infection according to the underlying disease.

Characteristic	Hematological Malignancies N = 369 (%)	Solid Tumors N = 98 (%)	Total N = 467 (%)	*p* Value
Age (years, mean, SD)	53.1 (15)	55.5 (13.3)		
Male sex	225 (61.1)	52 (53.1)	277 (59.4)	0.092
*Hematological malignancies* Acute leukemia Lymphoma Multiple myeloma Myelodysplastic syndrome Aplastic anemia Chronic myeloid leukemia Other ^1^ Hematopoietic stem cell transplant	207 (56) 84 (22.8) 49 (13.3) 8 (2.17) 8 (2.17) 8 (2.17) 5 (1.35) 142 (38.5)			
*Solid tumors* Breast cancer Colorectal cancer Head and Neck cancer Gynecological cancer Lung cancer Hepatobilliary tumor Other ^2^		24 (24.4) 23 (23.4) 11 (11.2) 10 (10.2) 9 (9.1) 5 (5.1) 16 (16.3)		
*Comorbidities* Diabetes mellitus Chronic obstructive pulmonary disease Chronic heart disease Chronic renal disease Chronic liver disease	92 (25) 38 (10.3) 21 (5.7) 18 (4.9) 11 (3) 8 (2.2)	36 (36.7) 14 (14.3) 3 (3.1) 6 (6.1) 3 (3.1) 6 (6.1)	128 (27.4) 52 (11.1) 24 (5.1) 24 (5.1) 14 (3) 14 (3)	0.020 0.265 0.295 0.620 1.00 0.086
*Immunosuppressive therapy* Previous chemotherapy (1 month) Previous corticosteroids	280 (76) 100 (27.1)	71 (72.4) 35 (36.1)	351 (75.2) 135 (29)	0.485 0.083
Neutropenia < 500cells/mm^3^	212 (57.6)	11 (11.2)	223 (48)	<0.001
High-risk MASCC index score	64 (28.7)	3 (25)	67 (28.5)	1.00
Parenteral nutrition	26 (7)	16 (16.3)	42 (9)	0.004
Previous antibiotic therapy (1 month)	196 (53.1)	39 (39.8)	235 (50.3)	0.019
Nosocomial acquisition	290 (78.6)	49 (50)	339 (72.6)	<0.001
*Type of catheter* Peripheral PICC Central venous catheter Port reservoir	37 (10) 21 (5.7) 306 (82.9) 4 (1.1)	23 (23.5) 12 (12.2) 18 (18.4) 44 (45)	60 (12.9) 33 (7.1) 324 (69.4) 48 (10.3)	0.001 0.024 <0.001 <0.001
*Etiology of CRBSI* Gram-positive Gram-negative Fungi MDRGNB	332 (90) 31 (8.7) 6 (1.6) 11 (3)	75 (76.5) 18 (18.4) 5 (5.1) 6 (6.1)	407 (87.2) 49 (10.5) 11 (2.36) 17 (3.6)	<0.001 0.004 0.058 0.141
Septic shock at presentation	18 (4.9)	6 (6.1)	24 (5.1)	0.620
Inadequate empirical antibiotic therapy	182 (49.6)	48 (49)	230 (49.5)	0.914
Catheter removal	289 (78.5)	89 (91.8)	378 (81.3)	0.003
Intensive care unit admission	20 (5.4)	3 (3.1)	23 (4.9)	0.438
*Case-fatality rates* Overall 30-days 7-days 48-h	46 (12.5) 18 (4.9) 7 (1.9)	20 (21.5) 6 (6.2) 1 (1)	66 (14.3) 24 (5.2) 8 (1.7)	0.027 0.608 1.00

Other ^1^: Chronic myelomonocytic leukemia (3), Chronic Lymphatic Leukemia (2). Other ^2^: Urinary tract (4), melanoma (3), timoma (3), esophageal cancer (2), sarcoma (2), central nervous system (2). MASCC: Multinational Association of Supportive Care in Cancer. CRBSI: catheter-related bloodstream infection. PICC: peripherally-inserted central catheter. MDRGNB: multidrug-resistant Gram-negative bacilli.

**Table 2:** Rates, etiology and type of catheter involved of all 467 episodes of catheter-related bloodstream infection through the study periods.

	2006–2010 N = 183 (39.2%)	2011–2015 N = 168 (36%)	2016–2020 N = 116 (24.8%)	Total N = 467 (%)	*p* Value
**Gram-positive** **Gram-negative** **Fungi** **MDRGNB**	171 (93.4) 10 (5.4) 2 (1.09) 4 (2.2)	151 (89.9) 13 (7.7) 4 (2.3) 3 (1.8)	85 (73.3) 26 (22.4) 5 (4.3) 10 (8.7)	407 (87.1) 49 (10.5) 11 (2.4) 17 (3.6)	<0.001 <0.001 0.076 0.008
**CVC** **Peripheral** **Port reservoir** **PICC**	125 (68.3) 41 (22.4) 12 (6.5) 5 (2.7)	120 (71.4) 18 (10.7) 16 (9.59 14 (8.3)	79 (68.1) 1 (0.86) 20 (17.2) 16 (13.8)	324 (69.3) 60 (12.4) 48 (10.2) 35 (7.5)	0.951 <0.001 0.004 <0.001

MDRGNB: multidrug-resistant Gram-negative bacilli; CVC: central venous catheter; PICC: peripherally-inserted central venous catheter.

**Table 3:** Etiology of all 467 episodes of catheter-related bloodstream infection.

	N	(%)
**GRAM-NEGATIVE**	**49**	**10.5**
*Pseudomonas aeruginosa*	14	3.0
*Klebsiella* spp.	11	2.4
*Enterobacter* spp.	7	1.5
*Escherichia coli*	5	1.1
*Stenotrophomonas maltophilia*	5	1.1
Other ^1^	7	1.3
MDRGNB ^2^	17	3.7
**GRAM-POSITIVE**	**407**	**87.1**
Coagulase-negative staphylococci	268	57.8
*Staphylococcus aureus*	106	22.8
*Methicillin-resistant S. aureus*	12	2.6
*Enterococcus faecium*	10	2.2
*Enterococcus faecalis*	9	1.9
*Corynebacterium* spp.	4	0.9
*Bacillus cereus*	3	0.6
Other ^3^	7	1.5
**FUNGI**	**11**	**2.4**
*Candida parapsilosis*	5	1.2
*Candida albicans*	2	0.4
*Candida krusei*	2	0.2
*Candida glabrata*	1	0.2
*Candida tropicalis*	1	0.2

^1^ Other: *Acinetobacter baumannii* (1), *Citrobacter freundi* (1), *Serratia marcescens* (1), *Providencia rettgeri* (1), *Ochrobactrum anthropi* (1), *Ralstonia picketti* (1), *Delfia acidovorans* (1). ^2^ MDRGN: multidrug-resistant Gram-negative bacilli. ^3^ Other: *Streptococcus pneumoniae* (1), *S. agalactiae* (1), *Oerkovia xanthineolyutica* (1), *Sarcinia* spp. (1), *Rothia mucilaginosa* (1), *Microbacterium testaceum* (1), *Micrococcus luteus* (1).

**Table 4:** Baseline characteristics and mortality of patients with Gram-negative catheter-related bloodstream infection compared to Gram-positive catheter-related bloodstream infection.

Characteristic	Gram-Positive N = 407 (%)	Gram-Negative N = 49 (%)	*p* Value
Age (years, mean, SD)	53.5 (±13.5)	53.7 (±14.3)	0.919
Male sex	244 (60)	28 (57.1)	0.82
Hematologic malignancy Solid tumor	323 (81.6) 75 (18.4)	31 (63.3) 18 (36.7)	**0.005**
HSCT (N = 140)	133 (32.7)	7 (14.3)	**0.013**
Comorbidities - Chronic kidney disease	106 (26) 8 (2)	17 (34.7) 5 (10.2)	0.26 **0.008**
Neutropenia	209 (51.4)	13 (27.1)	**0.002**
High risk MASCC score (N = 234)	58 (27.5)	8 (53.3)	**0.036**
Previous antibiotics (1 month)	203 (49.9)	24 (49.0)	1.00
Health-care acquisition	96 (23.6)	30 (61.2)	**<0.001**
Bacterial prophylaxis	26 (6.4)	1 (2)	0.34
Type of catheter - Peripheral - PICC - CVC - Port reservoir	54 (13.3) 32 (7.9) 288 (70.8) 33 (8.1)	2 (4.1) 2 (4.1) 32 (65.3) 13 (26.5)	0.064 0.56 0.53 **<0.001**
Hypotension (<80 mmHg)	34 (10.9)	9 (19.1)	0.169
Septic shock	20 (4.9)	4 (6.1)	0.72
Inadequate empirical antibiotic therapy ^a^ - Cephalosporins - β-lactam + β-lactamase inhibitors - Carbapenems - Monobactams - Aminoglycosides ^b^ - Quinolones - Glycopeptides	196 (48.4) 89 (45.4) 29 (14.9) 11 (5.6) 1 (0.5) 68 (34.7) 12 (6.1) 4 (2.0)	25 (51) 8 (32.0) 5 (20.0) 2 (8.0) 0 4 (16.0) 0 0	0.93
Case-fatality rates - At 48 hours - At 7 days - At 30 days	8 (2) 20 (5.2) 54 (13.5)	0 4 (8.2) 9 (18.4)	1.00 0.32 0.35

^a^ The number of empirical antibiotics exceed the number of catheter-related bloodstream infection episodes because some of the episodes were treated empirically with combination therapy. ^b^ Patients who received inadequate aminoglycoside empirical therapy were those who were already receiving a β-lactam regimen at fever onset, and the empirical antibiotic initiated at that point was an aminoglycoside, in order to broader the spectrum against Gram-negative bacilli. HSCT: hematopoietic stem cell transplant; MASCC: Multinational Association of Supportive Care in Cancer; PICC: peripherally-inserted catheter; CVC: central venous catheter.
